# Gradient-Based Multiple Robust Learning Calibration on Data Missing-Not-at-Random via Bi-Level Optimization

**DOI:** 10.3390/e27020196

**Published:** 2025-02-13

**Authors:** Shuxia Gong, Chen Ma

**Affiliations:** 1 Mogo Co., Ltd., Beijing 100000, China; 2 Gaoling School of Artificial Intelligence, Renmin University of China, Beijing 100872, China

**Keywords:** causal recommendation, multiple robust, calibration, bi-level optimization

## Abstract

Recommendation systems (RS) have become integral to numerous digital platforms and applications, ranging from e-commerce to content streaming field. A critical problem in RS is that the ratings are missing not at random (MNAR), which is due to the users always giving feedback on items with self-selection. The biased selection of rating data results in inaccurate rating prediction for all user-item pairs. Doubly robust (DR) learning has been studied in many tasks in RS, which is unbiased when either a single imputation or a single propensity model is accurate. In addition, multiple robust (MR) has been proposed with multiple imputation models and propensity models, and is unbiased when there exists a linear combination of these imputation models and propensity models is correct. However, we claim that the imputed errors and propensity scores are miscalibrated in the MR method. In this paper, we propose a gradient-based calibrated multiple robust learning method to enhance the debiasing performance and reliability of the rating prediction model. Specifically, we propose to use bi-level optimization to solve the weights and model coefficients of each propensity and imputation model in MR framework. Moreover, we adopt the differentiable expected calibration error as part of the objective to optimize the model calibration quality directly. Experiments on three real-world datasets show that our method outperforms the state-of-the-art baselines.

## 1. Introduction

Recommendation systems (RS) is an effective tool to address the problem of information overload and has been widely used in e-commerce, social media, and entertainment [1]. RS aims to predict user preferences for items based on collected historical interaction data [2,3]. However, the collected data cannot include all ratings from users to items, and the ratings are missing not at random (MNAR) due to users’ self-selection behavior, i.e., users can choose the item to rate freely, which is also known as selection bias problem [4,5]. The MNAR problem indicates that the collected dataset is not representative of the target population of interest (all user-item pairs), and the training distribution differs from the target test distribution. Ignoring such distributional shift will inevitably lead to sub-optimal recommendation performance [2,3,6]. To address the MNAR problem, one line of previous research proposed to use error imputation-based (EIB) methods, which first impute the missing ratings and then train the prediction model based on both observed and imputed ratings [6,7]. Additionally, another category of methods leverages propensity scores, which computes the probability of an event being observed, to reweight the observed ratings and align the distribution of observed data with the target population [3,8]. Furthermore, Doubly Robust (DR) method combines the error imputation and the inverse propensity re-weighting to achieve double robustness, which means the DR estimator achieves unbiasedness if either the imputed errors or the learned propensities are correct [2,9,10]. Furthermore, the Multiple Robust (MR) method is proposed to mitigate inaccuracies in single-model propensity scores or error imputations found in DR method [11]. By considering multiple candidate propensity and imputation models, MR estimator achieves unbiasedness if any of the propensity models, imputation models, or a linear combination of these models accurately estimate the true propensities or prediction errors.

However, we argue that the imputed errors and estimated propensity scores are miscalibrated in the existing MR method, which cannot reflect the ground-truth likelihood of the correctness of the true error or true propensity. For instance, if we have 100 user-item pairs with estimated propensity scores equal to 0.2, there should be exactly 20 ratings being observed and 80 ratings being unobserved. Although previous study has proposed to adopt calibration experts to calibrate the single propensity model and imputation model in DR estimator [12], this approach cannot be directly extended to the MR estimator, as calibrating each model individually is expensive and unreasonable due to the unbiasedness condition of MR in terms of linear combinations is not considered. Furthermore, the calibration metric previously used in [12] is non-differentiable and cannot be directly optimized.

To fill this gap, we propose the calibrated multiple robust learning (Cali-MR) method to calibrate the linear combinations of multiple imputation models and propensity models using bi-level optimization, which aims to learn an ensemble model that simultaneously possesses strong prediction performance and calibration ability. In this bi-level optimization, we adopt differentiable expected calibration errors to quantify the calibration ability that allows it to be directly optimized. The calibrated linear combination of propensity and imputation models is then used to train the prediction model based on a joint learning algorithm. The contributions of this paper are summarized as follows.

- We propose a novel MR calibration method using bi-level optimization via calibrating the ensemble imputation and propensity models and address the non-differentiable issue by adopting differentiable expected calibration errors.
- We further propose a bi-level calibrated multiple robust learning algorithm to update the calibrated imputation models and the prediction model. To the best of our knowledge, this is the first work to perform calibration for the MR estimator.
- We conduct extensive experiments on three real-world datasets, showing the effectiveness of our method compared to the state-of-the-art debiasing methods.

## 2. Related Works

### 2.1. Debiased Recommendation

The rating missing not at random (MNAR) problem results in the distribution of the observed population being different from the target population, hindering the prediction model from learning users’ true preferences [4,5,13,14,15]. There are many methods proposed to address this issue [16,17,18,19,20,21].

Specifically, methods including the Inverse Propensity Scoring (IPS), EIB, and DR were proposed to mitigate the MNAR problem in RS [6,22]. EIB methods might produce out-of-bound predictions; while the IPS method may suffer from large variance with small propensities [23]. DR methods combine the advantages of both the EIB and IPS methods, guaranteeing unbiasedness if either the error imputation model or propensity model is correctly specified.

There have been quantities of variants of DR methods to improve the debiasing performance, such as Multi-DR [24], BRD-DR [25], SDR [26], TDR [27], CDR [28], N-DR [29], DT-DR [30], UIDR [31], and OME-DR [32]. Besides, Multiple robust (MR) [11] combines multiple imputation models and propensity models, and is unbiased when there exists a linear combination of them is correct. In addition, Liu et al. [33] use an information bottleneck-based method and Yang et al. [34] and Wang et al. [35] use adversarial learning for debiasing. However, these methods fail to consider model calibration properties.

To mitigate this issue, DCE-DR [12] is proposed to calibrate the propensity and imputation model in the DR method. However, calibrating each imputation model and propensity model in MR is expensive and unreasonable, due to the unbiasedness condition of MR based on linear combinations is not taken into account. In this paper, we propose the Cali-MR method to calibrate multiple propensity and imputation models to further enhance the debiasing performance and reliability of MR method.

### 2.2. Model Calibration

Calibration means that the probability associated with the predicted class label should reflect its ground truth correctness likelihood [36,37], which plays an important role in building reliable, robust AI systems, especially in safety-critical fields such as medical diagnosis [38,39], self-driving [40,41], and financial decision making [42,43]. Early research demonstrated good calibration performance of simple neural networks on binary classification tasks [44]. However, with the rapid development of deep learning techniques, recent deep and complex neural networks are no longer well-calibrated [36].

**Figure 1:**
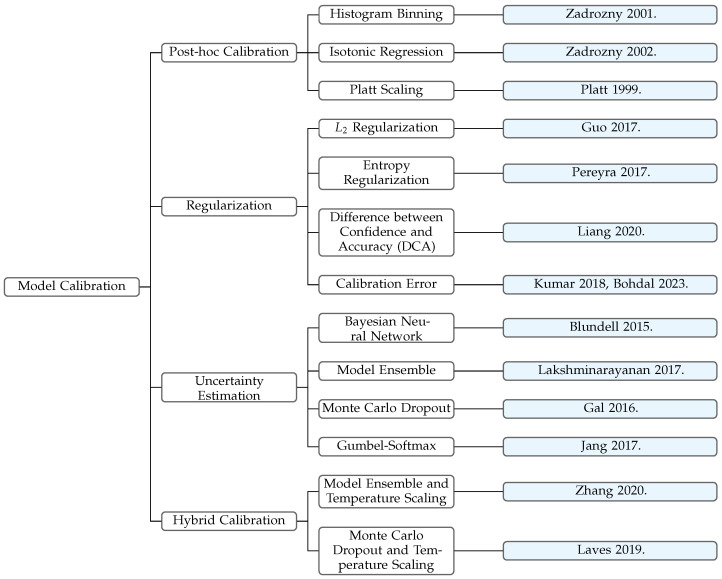
Categorization of model calibration methods: Post-hoc Calibration [46,47,48], Regularization [36,49,50,51,52], Uncertainty Estimation [53,54,55,56], and Hybrid Calibration [57,58].

Calibration methods can be divided into the following four categories [45]: post-hoc calibration, regularization methods, uncertainty estimation, and hybrid calibration methods. Post-hoc calibration methods aim to calibrate a model after training, including non-parametric calibration histogram binning [46], isotonic regression [47], and parametric methods such as Platt scaling [48]. Regularization methods adopt penalty terms such as the $L_{2}$ regularization [36], entropy regularization [49], difference between confidence and accuracy [50], and calibration errors [51,52] to ensure the calibration property. Uncertainty Estimation aims to alleviate model miscalibration by injecting randomness using Bayesian neural networks [53], model ensemble [54], Monte Carlo dropout [55], and Gumbel-softmax [56] based approaches. Hybrid calibration methods combine two or more methods to achieve calibration. For example, Zhang et al. [57] combines ensemble and temperature scaling and Laves et al. [58] adopts monte-carlo dropout with temperature scaling. We conclude the categorization of model calibration methods in Figure 1.

In this paper, we adopt a differentiable expected calibration error as part of the objective to ensure the model calibration. Compared to other techniques for calibration, our approach has the following advantages. First, unlike post-hoc methods that adjust calibration after training [47,48], our method based on calibration error regularization explicitly optimizes the calibration metric during the training process. Secondly, uncertainty estimation methods such as Monte Carlo Dropout typically rely on complex sampling processes [55], resulting in high computational costs, while hybrid calibration methods which combine multiple techniques, are similarly complex and challenging to implement [57,58]. By comparison, our proposed approach is both simple and computationally efficient. Furthermore, unlike other indirect regularization methods, such as $L_{2}$ or entropy regularization [36,49], our method directly incorporates differentiable expected calibration error as a loss function, enabling precise optimization of the calibration objective.

## 3. Preliminary

### 3.1. Debiased Recommendation

Let $U=\{u_{1},\cdots ,u_{m}\}$ be the users set, $I=\{i_{1},\cdots ,i_{n}\}$ be the item set, and D=U×I be the set of all user-item pairs. The rating matrix is denoted as $R\in R^{m\times n}$ with $r_{u,i}$ as element. Let $o_{u,i}\in \left\{0,1\right\}$ be the observation indicator, where $o_{u,i}=1$ indicates the rating $r_{u,i}$ is observed, otherwise is not. Define $x_{u,i}$ be the observed features. We denote the prediction model as $f_{\theta }\left(\cdot \right)$ parameterized by θ and the predicted ratings as ${\hat{r}}_{u,i}=f_{\theta }\left(x_{u,i}\right)$. The goal is to accurately predict $r_{u,i}$ for all user-item pairs, which can be achieved by minimizing the ideal loss

$$L_{\mathrm{ideal}}\left(\theta \right)=\frac{1}{\left|D\right|}\sum_{(u,i)\in D}L\left(f_{\theta }\left(x_{u,i}\right),r_{u,i}\right):=\frac{1}{\left|D\right|}\sum_{(u,i)\in D}e_{u,i},$$

where L(·,·) is the training loss function such as cross-entropy loss. However, in practice, we cannot obtain the complete rating matrix. We denote the set of user-item pairs with observed ratings as $O=\{\left(u,i\right)|o_{u,i}=1\}$. Thus, the naive method optimizes the average loss over the observed user-item pairs

$$L_{\mathrm{naive}}\left(\theta \right)=\frac{1}{\left|O\right|}\sum_{(u,i)\in O}e_{u,i}.$$

Due to the MNAR problem, $E\left[L_{\mathrm{naive}}\left(\theta \right)\right]\neq L_{\mathrm{ideal}}\left(\theta \right)$. Several methods were proposed to unbiasedly estimate the ideal loss, including the EIB, IPS, DR, and their variants. Because EIB and IPS can be regarded as special cases of DR, we only introduce the DR methods here. The loss function of the vanilla DR method is formulated as

$$L_{\mathrm{DR}}\left(\theta \right)=\frac{1}{\left|D\right|}\sum_{(u,i)\in D}\left[{\hat{e}}_{u,i}+\frac{o_{u,i}\left(e_{u,i}-{\hat{e}}_{u,i}\right)}{{\hat{p}}_{u,i}}\right],$$

where ${\hat{p}}_{u,i}≜\pi \left(x;\hat{\alpha }\right)$ is the estimation of propensity score $p_{u,i}=\mathrm{Pr}\left(o_{u,i}=1|x_{u,i}\right)$, and ${\hat{e}}_{u,i}=L\left(m\left(x_{u,i};\beta \right),{\hat{r}}_{u,i}\right)$ is the imputed error, while the imputation model is denoted as $m(x_{u,i};\hat{\beta })$. In addition, the multiple robust (MR) considers *J* propensity models $\pi_{1}\left(x;{\hat{\alpha }}_{1}\right),\cdots ,\pi_{J}\left(x;{\hat{\alpha }}_{J}\right)$ and *K* imputation models $m_{1}\left(x;{\hat{\beta }}_{1}\right),\cdots ,m_{K}\left(x;{\hat{\beta }}_{K}\right)$. Let ${\hat{p}}_{u,i}^{j}≜\pi_{j}\left(x_{u,i};{\hat{\alpha }}_{j}\right)$, ${\hat{m}}_{u,i}^{k}≜m_{k}\left(x;{\hat{\beta }}_{k}\right)$, the loss function of MR is shown below:

$$L_{\mathrm{MR}}\left(\theta \right)=\frac{1}{\left|D\right|}\sum_{(u,i)\in D}u^{T}\left(x_{u,i}\right)\cdot \hat{\eta }\left(\theta \right),$$

where $u\left(x_{u,i}\right)={\left(1/{\hat{p}}_{u,i}^{1},\cdots ,1/{\hat{p}}_{u,i}^{J},{\hat{m}}_{u,i}^{1},\cdots ,{\hat{m}}_{u,i}^{K}\right)}^{T}$ and $\hat{\eta }\left(\theta \right)$ is the solution by minimizing

$$\frac{1}{\left|D\right|}\sum_{(u,i)\in D}o_{u,i}{\left\{e_{u,i}-u^{T}\left(x_{u,i}\right)\cdot \eta \right\}}^{2}.$$

The MR estimator is unbiased when there exists a weight $W=(w_{1},w_{2},\ldots ,w_{J},0,0,\ldots ,0)$ satisfying $Wu\left(x_{u,i}\right)=1/p_{u,i}$ or $V=(0,\ldots ,0,v_{1},v_{2},\ldots ,v_{K})$ satisfying $Vu\left(x_{u,i}\right)=e_{u,i}$ for all user-item pairs.

### 3.2. Calibration

A model is calibrated if its output reflects the ground-truth likelihood of correctness [37]. For the propensity model $\pi (x;\hat{\alpha })$ and the observation indicator *o*, a formal definition is shown below:

$$E\left[o|\pi \left(x;\hat{\alpha }\right)=\hat{p}\right]=\hat{p}\;\forall \hat{p}\in \left[0,1\right].$$

For instance, if we have 100 samples with estimated propensity scores equal to 0.2, there should be exactly 20 samples being observed. Similarly, the formal definition for the calibrated imputation model $m(x;\hat{\beta })$ is formulated below:

$$E\left[e|m\left(x;\hat{\beta }\right)=\hat{e}\right]=\hat{e}\;\forall \hat{e}\in R.$$

To measure the miscalibration of the model, the Expected Calibration Error (ECE) metric is proposed [59]. For a propensity model $\pi (x;\hat{\alpha })$ and imputation model $m(x;\hat{\beta })$, the ECE is defined as follows:

$$\begin{array}{cc} \mathrm{ECE}(\hat{\alpha }) & =E_{\hat{p}}\left[\left|E\left[o|\pi \left(x;\hat{\alpha }\right)=\hat{p}\right]-\hat{p}\right|\right],\\ \mathrm{ECE}(\hat{\beta }) & =E_{\hat{e}}[|E\left[e|m\left(x;\hat{\beta }\right)=\hat{e}\right]-\hat{e}\left|\right]. \end{array}$$

## 4. Methodology

### 4.1. Distinctions from Previous Work

Previous studies have proposed to calibrate the single propensity model and imputation model in DR estimator [12], and they propose to use a binning strategy to estimate the ECE metric empirically, for example for the propensity model:

$$\hat{\mathrm{ECE}}\left(\hat{p}\right)=\sum_{m=1}^{M}\frac{\left|B_{m}\right|}{N}\left|\frac{\sum_{\left(u,i\right)\in B_{m}}o_{u,i}}{\left|B_{m}\right|}-\frac{\sum_{\left(u,i\right)\in B_{m}}{\hat{p}}_{u,i}}{\left|B_{m}\right|}\right|,$$

where $B_{m}$ is the predefined *m*-th bin and *N* is the corresponding number of samples in the bin.

However, how to properly calibrate multiple propensity and imputation models for MR estimators remains unexplored. A naive approach is individually calibrating each propensity and imputation model in the MR estimator. Note that this method is computationally expensive and overlooks the robust property of the MR estimator, that is, the MR estimator achieves unbiased if a linear combination of multiple candidate models is accurate.

Inspired by this, we propose to calibrate the linear combination of multiple models instead of calibrating each model individually. In addition, note that the previously used $\hat{\mathrm{ECE}}$ involves assigning each sample to a specific hard bin, making it non-differentiable and thus unsuitable for direct incorporation into the training objective. To address this issue, we employ a soft binning strategy to develop the differentiable expected calibration error metric and leverage it to construct a regularization term that constrains the model’s calibration error, which can be used for model training. Next, we will introduce the proposed Cali-MR in detail.

### 4.2. Differentiable Expected Calibration Error

To address the non-differentiable problem of $\hat{\mathrm{ECE}}$, we leverage the soft binning strategy [52], using the following differentiable expected calibration error (DECE) that allows directly optimize calibration quality to mitigate the model miscalibration. For example, the DECE for a propensity model $\pi (x;\hat{\alpha })$ is defined as:

$$\mathrm{DECE}\left(\hat{\alpha }\right)=\frac{1}{\left|D\right|}\sum_{m=1}^{M}\left|\sum_{(u,i)\in D}o_{m}\left(x_{u,i};\phi \right)\left(o_{u,i}-{\hat{p}}_{u,i}\right)\right|,$$

where $o_{m}\left(x_{u,i};\phi \right)=P\left(x_{u,i}\in B_{m}|{\hat{p}}_{u,i}\right)$ denotes the probability that how likely it is that ${\hat{p}}_{u,i}$ belongs to the m-th bin. In practice, the $o_{m}\left(x_{u,i};\phi \right)$ can be logistic regression or any other model [52].

In our Cali-MR, we adopt DECE as a regularization term and develop a gradient-based learning algorithm model training. Specifically, to calibrate the linear combination of multiple models, we formalize the propensity DECE loss $L_{\mathrm{DECE}}^{p}$ as follows:

$$\begin{array}{c} L_{\mathrm{DECE}}^{p}\left(w;\phi_{p};\alpha_{1},\ldots ,\alpha_{J}\right)=\frac{1}{\left|D\right|}\sum_{m=1}^{M}\left|\sum_{(u,i)\in D}o_{m}\left(x_{u,i};\phi_{p}\right)\left(o_{u,i}-\sum_{j=1}^{J}w_{j}{\hat{p}}_{u,i}^{j}\right)\right|, \end{array}$$

where $w=(w_{1},\cdots ,w_{J})$ is a given set of weight coefficients. This loss measures the calibration error of the combination model, and minimizing this loss improves the calibration ability of the current propensity model combination under the current combination coefficients. We use a one-layer neural network with softmax activation function to model the propensity soft binning model $o_{m}\left(x_{u,i};\phi_{p}\right)$ with parameters $\phi_{p}$, where *m* is a pre-defined hyper-parameter.

Similarly, the imputation DECE loss $L_{\mathrm{DECE}}^{e}$ under weight coefficients $v=(v_{1},\cdots ,v_{K})$ is formalized below:

$$\begin{array}{c} L_{\mathrm{DECE}}^{e}\left(v;\phi_{m};\beta_{1},\ldots ,\beta_{K}\right)=\frac{1}{\left|D\right|}\sum_{m=1}^{M}\left|\sum_{(u,i)\in D}o_{m}\left(x_{u,i};\phi_{m}\right)\left(\frac{o_{u,i}e_{u,i}}{\sum_{j=1}^{J}w_{j}{\hat{p}}_{u,i}^{j}}-\sum_{k=1}^{K}v_{k}{\hat{e}}_{u,i}^{k}\right)\right|, \end{array}$$

where we similarly model the imputation soft binning model $o_{m}\left(x_{u,i};\phi_{m}\right)$ with parameters $\phi_{m}$ with a one-layer neural network with softmax activation function. Note that the $e_{u,i}$ is missing for user-item pairs with $o_{u,i}=0$, we reweight the observed $e_{u,i}$ using the inverse of the linear combination of the multiple propensity models.

Based on the DECE loss $L_{\mathrm{DECE}}^{p}$ or $L_{\mathrm{DECE}}^{e}$, we can measure the calibration quality of given multiple models and weight coefficients, and further improve the calibration ability of the current combined model using a gradient-based algorithm by minimizing such differentiable loss.

### 4.3. Calibrated Multiple Robust Learning

Note that in calculating the DECE loss $L_{\mathrm{DECE}}^{p}$ or $L_{\mathrm{DECE}}^{e}$, the coefficients w or v need to be explicitly specified. However, these coefficients are unknown during the training process of the existing MR method. Therefore, we propose using a gradient-based bi-level optimization method to solve for the optimal coefficients, parameters of the soft binning model, and multiple imputation and propensity models. In addition, we alternatively update the prediction model and the calibrated imputation model based on a joint learning algorithm.

For propensity models, the optimization objective can be formalized as follows:

(1) $$\begin{array}{cc} \; & \left(\alpha_{1}^{*},\ldots ,\alpha_{J}^{*}\right)=\arg\min_{\alpha_{1},\ldots ,\alpha_{J}}\frac{1}{J}\sum_{j=1}^{J}L_{p_{j}}\left(\alpha_{j}\right)+\lambda L_{\mathrm{DECE}}^{p}\left(w^{*}\right)\\ \; & s.t.\;w^{*}\left(\alpha_{1},\ldots ,\alpha_{J}\right)=\arg\min_{w^{*}}L_{p}\left(w^{*}\left(\alpha_{1},\ldots ,\alpha_{J}\right)\right), \end{array}$$

where $L_{\mathrm{DECE}}^{p}\left(w\right)$ loss is the calibration constraints defined in Section 4.2, and $L_{p_{j}}\left(\alpha_{j}\right)$ is the training loss for a single propensity model $\pi_{J}\left(x;{\hat{\alpha }}_{J}\right)$ ensuring the accuracy of each independent propensity model, which is shown below:

$$\begin{array}{c} L_{p_{j}}\left(\alpha_{j}\right)=\frac{1}{\left|D\right|}\sum_{(u,i)\in D}\left[-o_{u,i}\cdot \log p_{u,i}^{j}-\left(1-o_{u,i}\right)\cdot \log\left(1-p_{u,i}^{j}\right)\right]. \end{array}$$

$L_{p}\left(w\left(\alpha_{1},\ldots ,\alpha_{J}\right)\right)$ is the loss for the combination coefficients w, aiming to learn a set of coefficients such that the linear combination $\sum_{j=1}^{J}w_{j}{\hat{p}}_{u,i}^{j}$ can accurately predict observation indicator $o_{u,i}$, which is shown below:

$$\begin{array}{c} L_{p}\left(w\left(\alpha_{1},\ldots ,\alpha_{J}\right)\right)=\frac{1}{\left|D\right|}\sum_{(u,i)\in D}\left[-o_{u,i}\cdot \log\left(\sum_{j=1}^{J}w_{j}{\hat{p}}_{u,i}^{j}\right)-\left(1-o_{u,i}\right)\cdot \log\left(1-\sum_{j=1}^{J}w_{j}{\hat{p}}_{u,i}^{j}\right)\right]. \end{array}$$

In this gradient-based bi-level optimization, we aim to train the propensity models such that each model performs well and their linear combination is well calibrated, where the coefficients also ensure the strong prediction performance of the combined model. For practical implementation, we first assumed update coefficients w through optimizing $L_{p}\left(w\right)$. Using these coefficients, we calculate the DECE loss $L_{\mathrm{DECE}}^{p}\left(w\right)$ and combine it with the base prediction loss of each propensity model denoted as $L_{p_{j}}\left(\alpha_{j}\right)$ to form the final loss. This final loss is then used to update the propensity models $\alpha_{1},\ldots ,\alpha_{J}$. After that, the loss $L_{p}\left(w\right)$ and $L_{\mathrm{DECE}}^{p}\left(w\right)$ are used to update the combination coefficients w and the soft binning model $\phi_{p}$ sequentially.

#### Multiple Imputation Calibration

With the calibrated propensity model $\sum_{j=1}^{J}w_{j}{\hat{p}}_{u,i}^{j}$ obtained from Equation (1) by the bi-level optimization, we can further calibrate the linear combination of multiple imputation models:

(2) $$\begin{array}{cc} \; & \left(\beta_{1}^{*},\ldots ,\beta_{K}^{*}\right)=\arg\min_{\beta_{1},\ldots ,\beta_{K}}\frac{1}{K}\sum_{k=1}^{K}L_{e_{k}}\left(\beta_{k}\right)+\lambda L_{\mathrm{DECE}}^{e}\left(v^{*}\right)\\ \; & s.t.\;v^{*}\left(\beta_{1},\ldots ,\beta_{K}\right)=\arg\min_{v^{*}}L_{e}\left(v^{*}\left(\beta_{1},\ldots ,\beta_{K}\right)\right), \end{array}$$

where $L_{\mathrm{DECE}}^{e}\left(v\right)$ is the calibration constraints shown in Section 4.2. The naive training loss for each imputation model $m_{K}\left(x;{\hat{\beta }}_{K}\right)$ is expressed as

$$\begin{array}{c} L_{e_{k}}\left(\beta_{k};\theta \right)=\frac{1}{\left|D\right|}\sum_{(u,i)\in D}\frac{o_{u,i}{\left(e_{u,i}-{\hat{e}}_{u,i}^{k}\right)}^{2}}{\sum_{j=1}^{J}w_{j}{\hat{p}}_{u,i}^{j}}, \end{array}$$

and the loss for the combination coefficients v is

$$\begin{array}{c} L_{e}\left(v\left(\beta_{1},\ldots ,\beta_{K}\right)\right)=\sum_{(u,i)\in D}\frac{o_{u,i}{\left(e_{u,i}-\sum_{k=1}^{K}v_{k}{\hat{e}}_{u,i}^{k}\right)}^{2}}{\left|D\right|\sum_{j=1}^{J}w_{j}{\hat{p}}_{u,i}^{j}}, \end{array}$$

which aims to learn a set of weight coefficients such that the linear combination of the imputation models $\sum_{k=1}^{K}v_{k}{\hat{e}}_{u,i}^{k}$ can unbiasedly estimate the prediction error $e_{u,i}$.

Similar to updating the propensity models, we first use $L_{e}\left(v\right)$ to assume update coefficients $v^{\prime }$. Based on that, we compute the DECE loss $L_{\mathrm{DECE}}^{e}\left(v^{\prime }\right)$, combine it with the base training loss of each imputation model $L_{e_{k}}\left(\beta_{k};\theta \right)$, and use the combined loss to update the imputation models $\beta_{1},\ldots ,\beta_{K}$. Then we adopt loss $L_{e}\left(v\right)$ and $L_{\mathrm{DECE}}^{e}\left(v\right)$ to update the combination coefficients v and the soft binning model $\phi_{e}$ sequentially. After obtaining the updated imputation models and coefficients, we jointly train the prediction model using the standard multiple robust learning algorithm. Specifically, we use ridge regression to calculate $\hat{\eta }$ in the MR estimator and update the prediction model based on the MR loss $L_{\mathrm{MR}}$ using different samples. We summarize the above gradient-based bi-level learning algorithm in Algorithm 1.
**Algorithm 1:** Gradient-Based Bi-level Calibrated Multiple Robust Learning
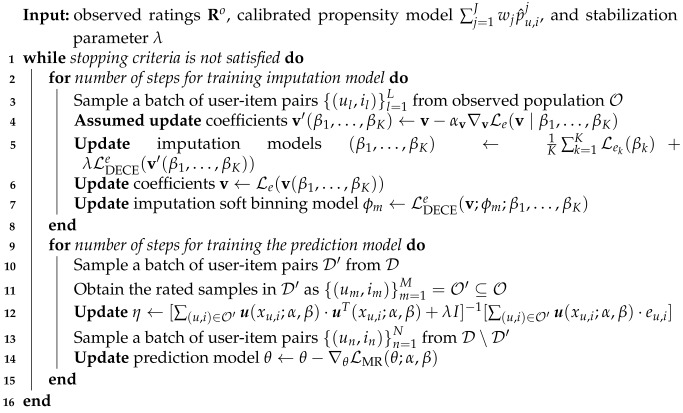


## 5. Experiments

### 5.1. Datasets

To evaluate the debiasing performance, we conduct experiments on three benchmark datasets **Coat** (https://www.cs.cornell.edu/~schnabts/mnar/, accessed on 15 January 2025) and **Yahoo! R3** (https://webscope.sandbox.yahoo.com), and **KuaiRec** (https://github.com/chongminggao/KuaiRec, accessed on 15 January 2025) [60], which are widely used in debiased RS with both missing not at random (MNAR) and missing at random (MAR) data. **Coat** dataset consists of 6960 MNAR training samples and 4640 MAR test samples derived from 290 users rating on 300 items. The **Yahoo! R3** dataset includes 311,704 MNAR training samples and 54,000 MAR test samples derived from 15,400 users rating on 1000 items. Both datasets are five-scale, and following previous works [61,62,63], we binarize the ratings greater than three to 1, and others to 0. The **KuaiRec** dataset is collected from a video-sharing platform and contains 4,676,570 video watching ratios derived from 1411 users evaluating 3327 videos. We binarize the continuous ratios greater than two to 1, otherwise to 0.

### 5.2. Baselines

We compare our method with the following baselines for comprehensive evaluations:

- **Naive** method [64] naively optimizes the average loss over the observed user-item pairs.
- **IPS** method [3] reweights the observed ratings with the inverse propensity scores.
- **SNIPS** method [65] reweights the observed ratings with self-normalized propensity scores to further reduce the variance.
- **ASIPS** method [22] generates reliable pseudo-ratings to mitigate propensity estimation bias and high variance problem.
- **DR** method [10] combines error imputation and inverse propensity reweighting to construct a doubly robust estimator, where imputed errors are typically set based on label prior knowledge, such as the mean value of the labels.
- **DR-JL** method [2] further proposes modeling error imputation with neural networks and jointly learns the prediction model and imputation model.
- **MRDR** method [66] enhances the **DR-JL** method by explicitly controlling the variance of the DR estimator through imputation model learning.
- **DR-BIAS** method [67] enhances the **DR-JL** method by further reducing the bias of the DR estimator through imputation model learning.
- **DR-MSE** method [67] further combines **MRDR** method and **DR-BIAS** method to achieve bias-variance trade-off and control the generalization error.
- **MR** method [11] adopts multiple candidate propensity and imputation models to mitigate inaccuracies in single-model propensity scores or error imputation in DR methods.
- **TDR** and **TDR-JL** methods [27] correct the imputed errors with targeted learning to reduce the bias and variance simultaneously for existing DR approaches
- **StableDR** method [26] constructs a stabilized DR estimator that has a weaker dependence on extrapolation and is robust to small propensities by learning constrained propensity scores.
- **IPS-V2** and **DR-V2** methods [68] learn the propensity model which can balance some manually selected functions such as the first and second moments of the features.
- **KBIPS** and **KBDR** methods [32] further propose to conduct causal balancing in the reproducing kernel Hilbert space (RKHS) and randomly select some kernel functions to balance for propensity model learning.
- **AKBIPS** and **AKBDR** methods [32] adaptively select the kernel functions which contribute the most to reducing the estimation bias to balance for propensity model learning.
- **DCE-DR** and **DCE-TDR** method [12] propose to calibrate the single propensity model and single imputation model in DR and TDR estimators through Mixture-of-Experts technique.

### 5.3. Experiment Protocols and Details

We evaluate the prediction performance using three widely adopted evaluation metrics: AUC (Area Under the ROC Curve), NDCG@*T* (Normalized Discounted Cumulative Gain), and F1@*T*.

- **AUC** [69] is a performance metric for classifiers that measures the probability of a randomly chosen positive example being ranked higher than a randomly chosen negative one. A higher AUC score reflects better ranking performance in differentiating positive instances from negative ones.
- **NDCG@*T*** [70] evaluates ranking performance by comparing the Discounted Cumulative Gain (DCG) of the top-*T* results to the Ideal DCG (IDCG), producing a normalized score between 0 and 1. A higher NDCG@*T* implies that more relevant items are ranked towards the top.

Let $r_{i}$ be the relevance of the item at rank *i*. We first compute the DCG at rank *T* as well as the IDCG@*T* by placing the most relevant items in the optimal (ideal) order:

$$\begin{array}{c} \mathrm{DCG}@T=\sum_{i=1}^{T}\frac{2^{r_{i}}-1}{\log_{2}\left(i+1\right)}\;,\;\mathrm{IDCG}@T=\sum_{i=1}^{T}\frac{2^{r_{i}^{*}}-1}{\log_{2}\left(i+1\right)}. \end{array}$$

where $r_{i}$ is the relevance of the *i*-th item at rank *i*, and $r_{i}^{*}$ denotes the relevance of the *i*-th item in the ideal ranking. NDCG@*T* is then defined as:

$$\begin{array}{c} \mathrm{NDCG}@T=\frac{\mathrm{DCG}@T}{\mathrm{IDCG}@T}. \end{array}$$

- **F1@*T*** [71] is the harmonic mean of precision and recall computed over the top-*T* predictions returned by a model. A higher F1@*T* indicates a better trade-off between precision and recall in the top-*T* results.

We set *T* = 5 on **Coat** and **Yahoo! R3**, and *T* = 20 on **KuaiRec**. In addition, we tune learning rate in {0.01,0.05} and weight decay in $\left\{1\times 10^{-6},5\times 10^{-6},1\times 10^{-5},\ldots ,1\times 10^{-3},5\times 10^{-3}\right\}$.We use the same hyperparameter search space and follow the results in Li et al. [32].

### 5.4. Performance Analysis

The experimental results are shown in Table 1, and we find that all the debiasing methods including both IPS-based and DR-based baselines outperform the Naive method, which demonstrates the importance of debiasing. Besides, among all baselines, AKBDR method introduces balancing kernel functions for propensity model training. This method adaptively identifies the most critical kernel functions to balance by fitting prediction errors, and well-trained propensity model effectively eliminates selection bias, thereby demonstrating strong performance on the Coat dataset. On the other hand, TDR introduces a targeted learning technique, which leverages propensity to enhance the error imputation, achieving reductions in both bias and variance. DCE-TDR further improves upon TDR by calibrating propensity model, utilizing the calibrated propensity for targeted learning, and achieving further performance gains. This calibration strategy enables DCE-TDR to deliver competitive results on the Yahoo! R3 and KuaiRec datasets. Furthermore, the proposed Cali-MR method exhibits superior overall performance and significantly outperforms existing methods on **Yahoo! R3** and **KuaiRec** datasets. This shows that calibrating multiple propensity and imputation models in a Multiple Robust estimator with weight coefficient learned in bi-level optimization further enhances the debiasing performance.

### 5.5. In-Depth Analysis

**Figure 2:**
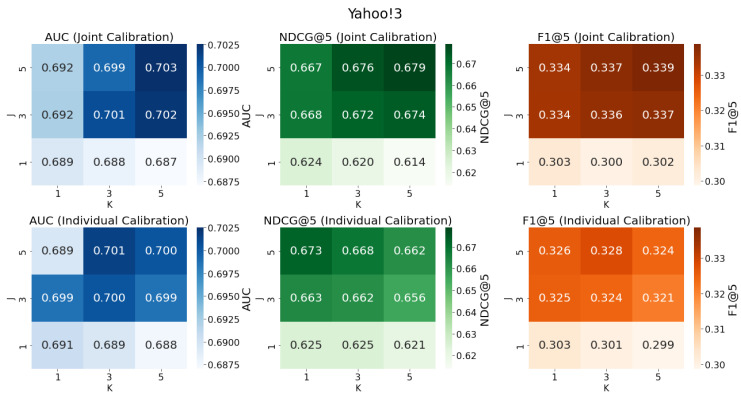
Comparison of joint calibration and individual calibration on the **Yahoo! R3** dataset, with different numbers of candidate propensity and imputation models.

**Figure 3:**
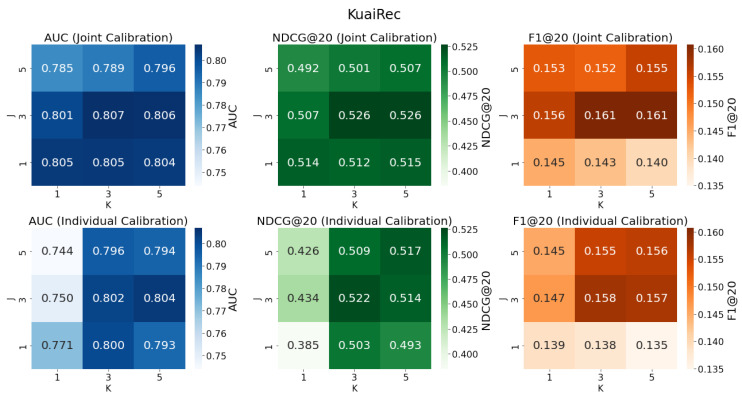
Comparison of joint calibration and individual calibration on the **KuaiRec** dataset, with different numbers of candidate propensity and imputation models.

We explore the difference between calibrating the ensemble model and calibrating individual models separately, with the number of propensity models *J* and imputation models *K* taking values from {1,3,5}. The results on the **KuaiRec** and **Yahoo! R3** datasets are shown in Figure 2 and Figure 3, where darker colors in the heatmap represent higher values of the corresponding evaluation metrics, indicating stronger model performance. The proposed method for calibrating the linear combination of multiple candidate models is referred to as ‛joint calibration’, while the approach of calibrating each individual candidate propensity model and then randomly selecting one calibrated propensity model to calibrate the candidate imputation models is referred to as ‛individual calibration’. We find that directly calibrating the ensemble model outperforms calibrating a single model individually, especially on the **Yahoo! R3** dataset. This demonstrates that the proposed Cali-MR method which takes into account the conditions for achieving unbiasedness in the MR estimator achieves better calibration and debiasing performance. On the other hand, examining the impact of the number of candidate models *J* and *K* on prediction performance, we find that when J=1 or K=1, i.e., when using only one propensity model or one imputation model, the prediction performance is poor, validating the effectiveness of incorporating more candidate models in the MR estimator. Additionally, the optimal (J,K) combinations for Cali-MR on **Yahoo! R3** and **KuaiRec** datasets are (5,5) and (3,3), respectively.

We conduct an ablation study of the proposed Cali-MR on three benchmark datasets, with the experimental results shown in Table 2. It can be observed that the prediction performance declines if either the propensity model or the imputation model is not calibrated, demonstrating the necessity of model calibration. Furthermore, the performance drop is more significant when removing the calibration of the propensity model compared to the imputation model, indicating that propensity models are more miscalibrated in the existing MR estimator. This highlights that the current propensity model learning approach, based on simple fitting to the observed variable indicator *o* with naive binary cross-entropy loss, fails to produce well-calibrated models, emphasizing the need to incorporate calibration loss on top of it.

**Figure 4:**
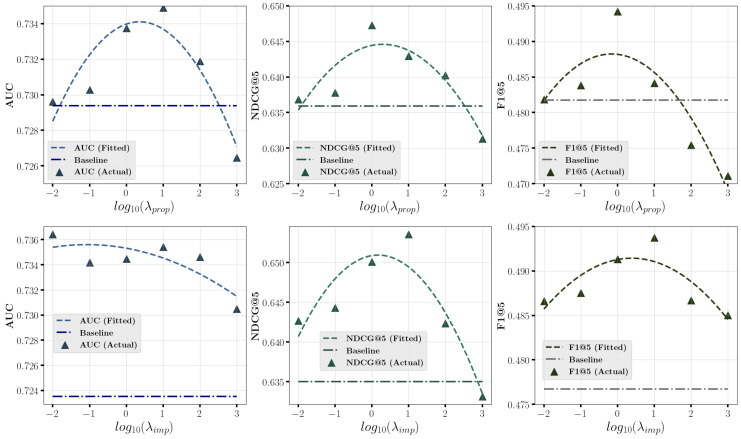
Impact of model calibration hyper-parameter $\lambda_{prop}$ in multiple propensity calibration and $\lambda_{imp}$ in multiple imputation calibration on **Coat** dataset.

**Figure 5:**
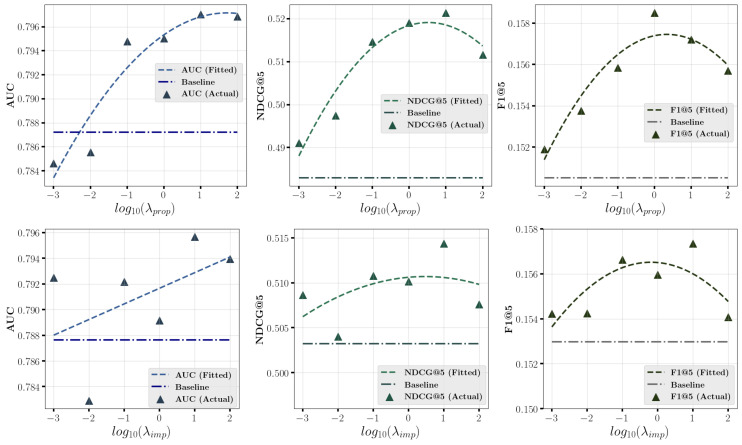
Impact of model calibration hyper-parameter $\lambda_{prop}$ in multiple propensity calibration and $\lambda_{imp}$ in multiple imputation calibration on **KuaiRec** dataset.

Figure 4 and Figure 5 investigate the impact of varying model calibration hyper-parameter λ in multiple propensity and imputation calibration on prediction performance on **Coat** and **KuaiRec** datasets. We record the results for different values of the hyper-parameter λ and fit a curve to show its variation trend, with a horizontal line representing the baseline method without calibration. We observe that when the hyper-parameter λ falls within an appropriate range, such as [0.1,100], the proposed method stably outperforms the baseline method, with the best performance achieved around moderate values, such as λ=10. Notably, on the **Coat** dataset, when the value of hyperparameter λ is set too large (e.g., 1000), the model fails to achieve good prediction performance, which indicates the propensity and imputation models overly emphasize calibration at the expense of prediction accuracy, leading to poor performance. This highlights the need to balance the prediction and calibration capabilities of propensity and imputation models during training.

**Figure 6:**
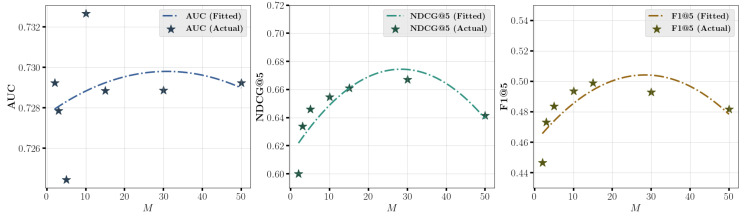
Effect of varying *M* in soft binning strategy on prediction performance on **Coat** dataset.

**Figure 7:**
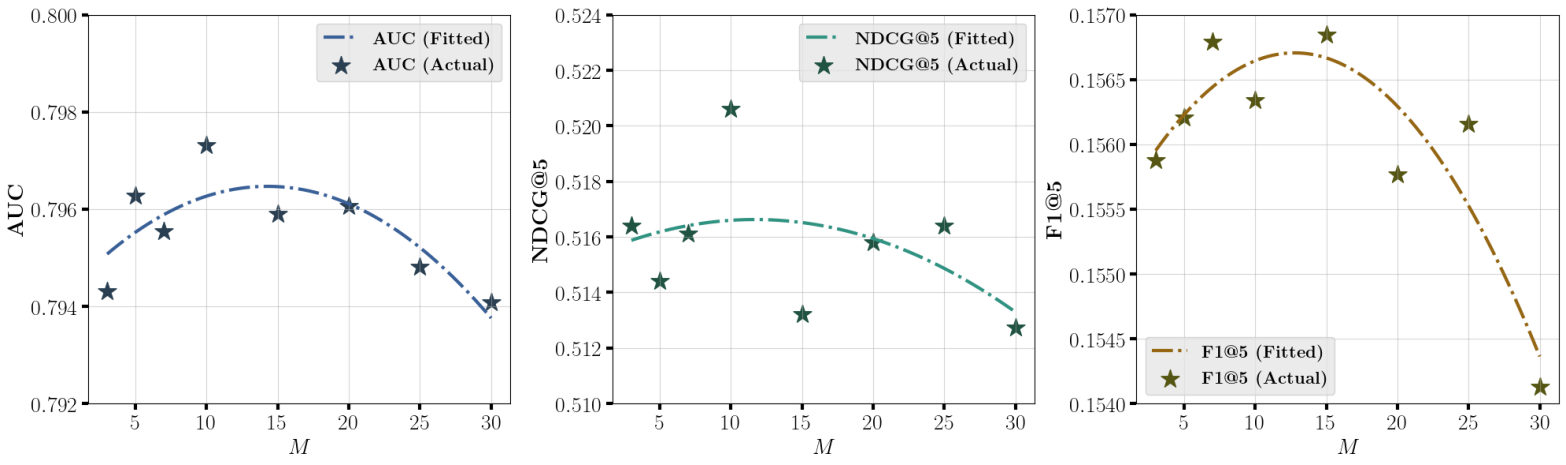
Effect of varying *M* in soft binning strategy on prediction performance on **KuaiRec** dataset.

Figure 6 and Figure 7 explore the effect of varying number of bins *M* in soft binning strategy on prediction performance on **Coat** and **KuaiRec** datasets. The scatter points represent the results for different values of *M*, and a fitted curve illustrates the trend of predictive performance as *M* changes. The results in the figure indicate that the optimal performance is achieved at moderate values of *M*, such as around 30 for **Coat** dataset and 15 for **KuaiRec** dataset. However, performance declines when *M* is either too large or too small. A possible reason is that when *M* is too small, the probability of samples with different prediction values ($\hat{p}$ or $\hat{e}$) being assigned to the same bin increases. Conversely, when *M* is too large, the probability of samples with similar prediction values ($\hat{p}$ or $\hat{e}$) being assigned to the same bin decreases. Both situations can lead to inaccurate DECE estimation, thereby reducing the calibration quality of the model.

The results in Table 3 demonstrate that proposed Cali-MR achieves comparable ECE performance to the current SOTA model, DCE-TDR. However, when evaluating the relative ECE reduction compared to the base models, we observe that DCE-TDR achieves a smaller relative improvement over TDR because TDR itself, leveraging targeted learning, already exhibits a relatively calibrated imputation model. Conversely, the original MR model shows a high ECE, indicating poor calibration, while the proposed Cali-MR significantly reduces the ECE, thereby enhancing calibration quality, which validates the effectiveness of the proposed approach.

We compare the training time and parameter size of different methods on Table 4. The experimental results show that the Naive method, which adopts a single model to fit observed ratings, has a parameter size of 1×. The IPS method introduces an additional propensity model, resulting in a parameter size of 2×, while the DR method further introduces an imputation model, with a parameter size of 3×. In this experiment, the MR method adopts two candidate propensity and imputation models, leading to a parameter size of 5×. As an improvement, our proposed Cali-MR (J = 2, K = 2) adds only a soft binning module, which increases the parameter count marginally compared to the original MR method, thus still maintaining a parameter size of 5×. In other words, the proposed method achieves significant performance improvements over the existing MR method, while maintaining the same parameter size. Additionally, by comparing the training times for MR and Cali-MR (J = 2, K = 2), we observe that model calibration does not add substantial training time, for example, with times of 127.8 and 144.56 on **Yahoo! R3** dataset, respectively. Furthermore, increasing the number of candidate models (J and K) for Cali-MR does not lead to a significant increase in training time. This is because as the number of candidate models increases, the ensemble model tends to overfit, and due to early stopping strategy, the number of epochs required for convergence varies with the number of candidate models. In summary, compared to the MR method, the proposed Cali-MR method improves model calibration and prediction accuracy with almost the same parameter size and acceptable training time.

## 6. Conclusions

In this paper, we explore how to properly calibrate multiple propensity and imputation models in a Multiple Robust (MR) estimator. First, we argue that calibrating each candidate model individually is too costly and unreasonable due to the unbiasedness condition of MR in terms of linear combinations is not considered. Based on this, we propose using a gradient-based bi-level optimization method to calibrate the linear combination of multiple candidate models. Specifically, in the bi-level optimization, we first assumed to update the combination coefficients to obtain the best-performing combination coefficients under the current candidate model parameters. Then, based on these coefficients, we update the candidate model parameters to ensure that each model maintains good prediction performance, while also ensuring that the combination model achieves strong calibration ability, using the differentiable expected calibration error metric that allows it to be directly optimized. Experimental results on three real-world datasets demonstrate that the proposed multiple propensity and imputation calibration method further enhances the prediction performance.

Regarding the broader impact, this is the first work to address the calibration of MR models, by introducing the differentiable expected calibration error (DECE) to directly optimize the objective, rather than relying on post-hoc adjustments in previous work. This provides a novel perspective on model calibration within the debiasing recommendation field. The main limitation of this work lies in the use of bi-level optimization to update parameters of multiple candidate models and the optimal combination coefficients within the MR method. This results in longer training times for the proposed Cali-MR method compared to the original MR method. A potential future work is to develop an alternative optimization algorithm to reduce the computational cost. Furthermore, in future works, we plan to investigate whether other calibration metrics, aside from ECE, might be more suitable for debiasing recommendation scenarios. 

## Figures and Tables

**Table 1:** Performance on AUC, NDCG@*T*, and F1@*T* on **Coat**, **Yahoo! R3** and **KuaiRec**. The best and the second best results are bolded and underlined, where * means statistically significant results (p-value≤0.05) using the paired-*t*-test.

	Coat			Yahoo! R3			KuaiRec		
**Method**	**AUC**	**NDCG@5**	**F1@5**	**AUC**	**NDCG@5**	**F1@5**	**AUC**	**NDCG@20**	**F1@20**
Naive	0.$703_{\pm 0.006}$	0.$605_{\pm 0.012}$	0.$467_{\pm 0.007}$	0.$673_{\pm 0.001}$	0.$635_{\pm 0.002}$	0.$306_{\pm 0.002}$	0.$753_{\pm 0.001}$	0.$449_{\pm 0.002}$	0.$124_{\pm 0.002}$
IPS	0.$717_{\pm 0.007}$	0.$617_{\pm 0.009}$	0.$473_{\pm 0.008}$	0.$678_{\pm 0.001}$	0.$638_{\pm 0.002}$	0.$318_{\pm 0.002}$	0.$755_{\pm 0.004}$	0.$452_{\pm 0.010}$	0.$131_{\pm 0.004}$
SNIPS	0.$714_{\pm 0.012}$	0.$614_{\pm 0.012}$	0.$474_{\pm 0.009}$	0.$683_{\pm 0.002}$	0.$639_{\pm 0.002}$	0.$316_{\pm 0.002}$	0.$754_{\pm 0.003}$	0.$453_{\pm 0.004}$	0.$126_{\pm 0.003}$
ASIPS	0.$719_{\pm 0.009}$	0.$618_{\pm 0.012}$	0.$476_{\pm 0.009}$	0.$679_{\pm 0.003}$	0.$640_{\pm 0.003}$	0.$319_{\pm 0.003}$	0.$757_{\pm 0.005}$	0.$474_{\pm 0.007}$	0.$130_{\pm 0.005}$
IPS-V2	0.$726_{\pm 0.005}$	0.$627_{\pm 0.009}$	0.$479_{\pm 0.008}$	0.$685_{\pm 0.002}$	0.$646_{\pm 0.003}$	0.$320_{\pm 0.002}$	0.$764_{\pm 0.001}$	0.$476_{\pm 0.003}$	0.$135_{\pm 0.003}$
KBIPS	0.$714_{\pm 0.003}$	0.$618_{\pm 0.010}$	0.$474_{\pm 0.007}$	0.$676_{\pm 0.002}$	0.$642_{\pm 0.003}$	0.$318_{\pm 0.002}$	0.$763_{\pm 0.001}$	0.$463_{\pm 0.007}$	0.$134_{\pm 0.002}$
AKBIPS	0.$732_{\pm 0.004}$	0.$636_{\pm 0.006}$	0.$483_{\pm 0.006}$	0.$689_{\pm 0.001}$	0.$658_{\pm 0.002}$	0.$324_{\pm 0.002}$	0.$766_{\pm 0.003}$	0.$478_{\pm 0.009}$	0.$138_{\pm 0.003}$
DR	0.$718_{\pm 0.008}$	0.$623_{\pm 0.009}$	0.$474_{\pm 0.007}$	0.$684_{\pm 0.002}$	0.$658_{\pm 0.003}$	0.$326_{\pm 0.002}$	0.$755_{\pm 0.008}$	0.$462_{\pm 0.010}$	0.$135_{\pm 0.005}$
DR-JL	0.$723_{\pm 0.005}$	0.$629_{\pm 0.007}$	0.$479_{\pm 0.005}$	0.$685_{\pm 0.002}$	0.$653_{\pm 0.002}$	0.$324_{\pm 0.002}$	0.$766_{\pm 0.002}$	0.$467_{\pm 0.005}$	0.$136_{\pm 0.003}$
MRDR-JL	0.$727_{\pm 0.005}$	0.$627_{\pm 0.008}$	0.$480_{\pm 0.008}$	0.$684_{\pm 0.002}$	0.$652_{\pm 0.003}$	0.$325_{\pm 0.002}$	0.$768_{\pm 0.005}$	0.$473_{\pm 0.007}$	0.$139_{\pm 0.004}$
DR-BIAS	0.$726_{\pm 0.004}$	0.$629_{\pm 0.009}$	0.$482_{\pm 0.007}$	0.$685_{\pm 0.002}$	0.$653_{\pm 0.002}$	0.$325_{\pm 0.003}$	0.$768_{\pm 0.003}$	0.$477_{\pm 0.006}$	0.$137_{\pm 0.004}$
DR-MSE	0.$727_{\pm 0.007}$	0.$631_{\pm 0.008}$	0.$484_{\pm 0.007}$	0.$687_{\pm 0.002}$	0.$657_{\pm 0.003}$	0.$327_{\pm 0.003}$	0.$770_{\pm 0.003}$	0.$480_{\pm 0.006}$	0.$140_{\pm 0.003}$
MR	0.$724_{\pm 0.004}$	0.$636_{\pm 0.006}$	0.$481_{\pm 0.006}$	0.$691_{\pm 0.002}$	0.$647_{\pm 0.002}$	0.$316_{\pm 0.003}$	0.$776_{\pm 0.005}$	0.$483_{\pm 0.006}$	0.$142_{\pm 0.003}$
TDR	0.$714_{\pm 0.006}$	0.$634_{\pm 0.011}$	0.$483_{\pm 0.008}$	0.$688_{\pm 0.003}$	0.$662_{\pm 0.002}$	0.$329_{\pm 0.002}$	0.$772_{\pm 0.003}$	0.$486_{\pm 0.005}$	0.$140_{\pm 0.003}$
TDR-JL	0.$731_{\pm 0.005}$	0.$639_{\pm 0.007}$	0.$484_{\pm 0.007}$	0.$689_{\pm 0.002}$	0.$656_{\pm 0.004}$	0.$327_{\pm 0.003}$	0.$772_{\pm 0.003}$	0.$489_{\pm 0.005}$	0.$142_{\pm 0.003}$
StableDR	0.$735_{\pm 0.005}$	0.$640_{\pm 0.007}$	0.$484_{\pm 0.006}$	0.$688_{\pm 0.002}$	0.$661_{\pm 0.003}$	0.$329_{\pm 0.002}$	0.$773_{\pm 0.001}$	0.$491_{\pm 0.003}$	0.$143_{\pm 0.003}$
DR-V2	0.$734_{\pm 0.007}$	0.$639_{\pm 0.009}$	0.$487_{\pm 0.006}$	0.$690_{\pm 0.002}$	0.$660_{\pm 0.005}$	0.$328_{\pm 0.002}$	0.$773_{\pm 0.003}$	0.$488_{\pm 0.006}$	0.$142_{\pm 0.004}$
KBDR	0.$730_{\pm 0.003}$	0.$631_{\pm 0.005}$	0.$482_{\pm 0.006}$	0.$682_{\pm 0.002}$	0.$648_{\pm 0.003}$	0.$323_{\pm 0.002}$	0.$765_{\pm 0.004}$	0.$460_{\pm 0.006}$	0.$138_{\pm 0.003}$
AKBDR	$0.745_{\pm 0.004}$	0.$645_{\pm 0.008}$	0.493_±0.007_	0.$692_{\pm 0.002}$	0.$661_{\pm 0.002}$	0.$328_{\pm 0.002}$	0.$782_{\pm 0.003}$	0.$498_{\pm 0.008}$	0.$147_{\pm 0.003}$
DCE-DR	0.$736_{\pm 0.006}$	0.$648_{\pm 0.007}$	0.$489_{\pm 0.005}$	0.$698_{\pm 0.002}$	0.$670_{\pm 0.002}$	0.333_±0.003_	0.$795_{\pm 0.004}$	0.$512_{\pm 0.005}$	0.$153_{\pm 0.002}$
DCE-TDR	0.$740_{\pm 0.004}$	0.651_±0.006_	0.$489_{\pm 0.007}$	0.701_±0.002_	0.672_±0.002_	0.$331_{\pm 0.002}$	$0.798_{\pm 0.005}$	0.514_±0.006_	0.155_±0.002_
Cali-MR	$0.741_{\pm 0.002}$	$0.658_{\pm 0.004}^{*}$	$0.495_{\pm 0.004}$	$0.703_{\pm 0.002}^{*}$	$0.678_{\pm 0.002}^{*}$	$0.338_{\pm 0.004}^{*}$	$0.798_{\pm 0.003}$	$0.521_{\pm 0.005}^{*}$	$0.158_{\pm 0.002}^{*}$

**Table 2:** Ablation study of Cali-MR on **Coat**, **Yahoo! R3** and **KuaiRec** datasets.

Method	Coat			Yahoo! R3			KuaiRec		
	AUC	NDCG@5	F1@5	AUC	NDCG@5	F1@5	AUC	NDCG@20	F1@20
Cali-MR	0.741	0.658	0.495	0.703	0.678	0.338	0.798	0.521	0.158
Cali-MR w/o prop	0.730	0.640	0.486	0.700	0.674	0.336	0.787	0.483	0.151
Cali-MR w/o imp	0.736	0.639	0.483	0.703	0.674	0.336	0.793	0.509	0.155
Cali-MR w/o imp & prop	0.727	0.635	0.477	0.698	0.667	0.331	0.783	0.482	0.148

**Table 3:** Comparison of Expected Calibration Error (ECE) on **Coat**, **Yahoo! R3** and **KuaiRec** datasets, where RD refers to the relative decrease value over the corresponding base model.

	Coat		Yahoo! R3		KuaiRec	
Method	ECE	RD	ECE	RD	ECE	RD
DR-JL	0.1626	-	0.0589	-	0.0999	-
DCE-DR	0.1428	0.0198	0.0554	0.0035	0.0451	0.0548
TDR-JL	0.1468	-	0.0481	-	0.0499	-
DCE-TDR	0.1270	0.0198	0.0476	0.0005	0.0488	0.0011
MR	0.1512	-	0.1594	-	0.2367	-
Cali-MR	0.1239	0.0273	0.0349	0.1245	0.0519	0.1848

**Table 4:** Comparison of training time (seconds) and parameter size (Naive method denoted as 1×) on **Coat**, **Yahoo! R3** and **KuaiRec** datasets.

Method	Coat		Yahoo! R3		KuaiRec	
	Time	Params	Time	Params	Times	Params
Naive	4.04	1×	26.07	1×	11.67	1×
IPS	6.54	2×	32.84	2×	15.14	2×
DR	17.41	3×	43.18	3×	31.20	3×
DR-JL	18.81	3×	166.21	3×	110.01	3×
TDR-JL	21.13	3×	128.88	3×	101.09	3×
MR (J = 2, K = 2)	13.55	5×	127.8	5×	114.78	5×
Cali-DR (J = 1, K = 1)	21.03	3×	132.98	3×	128.31	3×
Cali-MR (J = 2, K = 2)	23.28	5×	144.56	5×	124.58	5×
Cali-MR (J = 1, K = 5)	21.57	5×	139.80	5×	130.34	5×
Cali-MR (J = 1, K = 5)	21.43	7×	136.77	7×	141.46	7×
Cali-MR (J = 3, K = 3)	19.55	5×	127.80	5×	106.66	7×
Cali-MR (J = 3, K = 3)	25.43	7×	151.97	7×	127.47	7×
Cali-MR (J = 3, K = 5)	23.59	9×	166.42	9×	163.02	9×
Cali-MR (J = 5, K = 3)	23.71	9×	143.76	9×	103.60	9×
Cali-MR (J = 5, K = 3)	29.35	9×	132.74	9×	128.03	9×
Cali-MR (J = 5, K = 5)	25.65	11×	153.79	11×	166.73	11×

## Data Availability

Restrictions apply to the availability of these data. Our code is available at https://github.com/Yilu114/Cali-MR (accessed on 15 January 2025). Data were obtained from GitHub and are available https://github.com/RyanWangZf/CVIB-Rec with the permission of GitHub (accessed on 15 January 2025).

## References

[1] Ricci F., Rokach L., Shapira B. (2010). Introduction to recommender systems handbook. Recommender Systems Handbook.

[2] Wang X., Zhang R., Sun Y., Qi J. Doubly Robust Joint Learning for Recommendation on Data Missing Not at Random. Proceedings of the ICML.

[3] Schnabel T., Swaminathan A., Singh A., Chandak N., Joachims T. Recommendations as Treatments: Debiasing Learning and Evaluation. Proceedings of the ICML.

[4] Chen J., Dong H., Wang X., Feng F., Wang M., He X. (2022). Bias and Debias in Recommender System: A Survey and Future Directions. Acm Trans. Inf. Syst..

[5] Wu P., Li H., Deng Y., Hu W., Dai Q., Dong Z., Sun J., Zhang R., Zhou X.H. On the Opportunity of Causal Learning in Recommendation Systems: Foundation, Estimation, Prediction and Challenges. Proceedings of the IJCAI.

[6] Steck H. Training and testing of recommender systems on data missing not at random. Proceedings of the KDD.

[7] Chang Y.W., Hsieh C.J., Chang K.W., Ringgaard M., Lin C.J. (2010). Training and testing low-degree polynomial data mappings via linear SVM. J. Mach. Learn. Res..

[8] Saito Y., Yaginuma S., Nishino Y., Sakata H., Nakata K. Unbiased recommender learning from missing-not-at-random implicit feedback. Proceedings of the WSDM.

[9] Morgan S.L., Winship C. (2015). Counterfactuals and Causal Inference: Methods and Principles for Social Research.

[10] Saito Y. Doubly robust estimator for ranking metrics with post-click conversions. Proceedings of the RecSys.

[11] Li H., Dai Q., Li Y., Lyu Y., Dong Z., Zhou X.H., Wu P. Multiple Robust Learning for Recommendation. Proceedings of the AAAI.

[12] Kweon W., Yu H. Doubly Calibrated Estimator for Recommendation on Data Missing Not At Random. Proceedings of the WWW.

[13] Luo H., Zhuang F., Xie R., Zhu H., Wang D., An Z., Xu Y. (2024). A survey on causal inference for recommendation. Innovation.

[14] Li M., Sui H. Debiased Recommendation via Machine Unlearning. Proceedings of the AAAI Workshop on Artificial Intelligence with Causal Techniques.

[15] Wang W., Zhang Y., Li H., Wu P., Feng F., He X. Causal Recommendation: Progresses and Future Directions. Proceedings of the SIGIR.

[16] Saito Y., Nomura M. Towards Resolving Propensity Contradiction in Offline Recommender Learning. Proceedings of the IJCAI.

[17] Wang H., Yang W., Yang L., Wu A., Xu L., Ren J., Wu F., Kuang K. Estimating Individualized Causal Effect with Confounded Instruments. Proceedings of the KDD.

[18] Zou H., Wang H., Xu R., Li B., Pei J., Jian Y.J., Cui P. Factual Observation Based Heterogeneity Learning for Counterfactual Prediction. Proceedings of the CCLR.

[19] Wang H., Kuang K., Lan L., Wang Z., Huang W., Wu F., Yang W. (2024). Out-of-distribution generalization with causal feature separation. IEEE Trans. Knowl. Data Eng..

[20] Wang H., Kuang K., Chi H., Yang L., Geng M., Huang W., Yang W. Treatment effect estimation with adjustment feature selection. Proceedings of the KDD.

[21] Wu A., Kuang K., Xiong R., Li B., Wu F. Stable estimation of heterogeneous treatment effects. Proceedings of the ICML.

[22] Saito Y. Asymmetric Tri-training for Debiasing Missing-Not-At-Random Explicit Feedback. Proceedings of the SIGIR.

[23] Wang H., Chang T.W., Liu T., Huang J., Chen Z., Yu C., Li R., Chu W. Escm2: Entire space counterfactual multi-task model for post-click conversion rate estimation. Proceedings of the SIGIR.

[24] Zhang W., Bao W., Liu X.Y., Yang K., Lin Q., Wen H., Ramezani R. Large-scale Causal Approaches to Debiasing Post-click Conversion Rate Estimation with Multi-task Learning. Proceedings of the WWW.

[25] Ding S., Wu P., Feng F., He X., Wang Y., Liao Y., Zhang Y. Addressing Unmeasured Confounder for Recommendation with Sensitivity Analysis. Proceedings of the KDD.

[26] Li H., Zheng C., Wu P. StableDR: Stabilized Doubly Robust Learning for Recommendation on Data Missing Not at Random. Proceedings of the ICLR.

[27] Li H., Lyu Y., Zheng C., Wu P. TDR-CL: Targeted Doubly Robust Collaborative Learning for Debiased Recommendations. Proceedings of the ICLR.

[28] Song Z., Chen J., Zhou S., Shi Q., Feng Y., Chen C., Wang C. CDR: Conservative Doubly Robust Learning for Debiased Recommendation. Proceedings of the CIKM.

[29] Li H., Zheng C., Ding S., Feng F., He X., Geng Z., Wu P. Be Aware of the Neighborhood Effect: Modeling Selection Bias under Interference for Recommendation. Proceedings of the ICLR.

[30] Zhang H., Wang S., Li H., Zheng C., Chen X., Liu L., Luo S., Wu P. Uncovering the Propensity Identification Problem in Debiased Recommendations. Proceedings of the ICDE.

[31] Li H., Zheng C., Wang S., Wu K., Wang E., Wu P., Geng Z., Chen X., Zhou X.H. Relaxing the Accurate Imputation Assumption in Doubly Robust Learning for Debiased Collaborative Filtering. Proceedings of the ICML.

[32] Li H., Zheng C., Xiao Y., Wu P., Geng Z., Chen X., Cui P. Debiased collaborative filtering with kernel-based causal balancing. Proceedings of the ICLR.

[33] Liu D., Cheng P., Zhu H., Dong Z., He X., Pan W., Ming Z. (2023). Debiased representation learning in recommendation via information bottleneck. ACM Trans. Recomm. Syst..

[34] Yang M., Dai Q., Dong Z., Chen X., He X., Wang J. Top-n recommendation with counterfactual user preference simulation. Proceedings of the CIKM.

[35] Wang J., Li H., Zhang C., Liang D., Yu E., Ou W., Wang W. Counterclr: Counterfactual contrastive learning with non-random missing data in recommendation. Proceedings of the ICDM.

[36] Guo C., Pleiss G., Sun Y., Weinberger K.Q. On calibration of modern neural networks. Proceedings of the ICML.

[37] Kull M., Silva Filho T., Flach P. Beta calibration: A well-founded and easily implemented improvement on logistic calibration for binary classifiers. Proceedings of the AISTATS.

[38] Caruana R., Lou Y., Gehrke J., Koch P., Sturm M., Elhadad N. Intelligible models for healthcare: Predicting pneumonia risk and hospital 30-day readmission. Proceedings of the KDD.

[39] Huang Y., Li W., Macheret F., Gabriel R.A., Ohno-Machado L. (2020). A tutorial on calibration measurements and calibration models for clinical prediction models. J. Am. Med. Inform. Assoc..

[40] Bojarski M. (2016). End to end learning for self-driving cars. arXiv.

[41] Chen Z., Huang X. End-to-end learning for lane keeping of self-driving cars. Proceedings of the 2017 IEEE Intelligent Vehicles Symposium (IV).

[42] Büchel P., Kratochwil M., Nagl M., Rösch D. (2022). Deep calibration of financial models: Turning theory into practice. Rev. Deriv. Res..

[43] Biagini F., Gonon L., Walter N. (2024). Approximation rates for deep calibration of (rough) stochastic volatility models. SIAM J. Financ. Math..

[44] Niculescu-Mizil A., Caruana R. Predicting good probabilities with supervised learning. Proceedings of the ICML.

[45] Wang C. (2023). Calibration in deep learning: A survey of the state-of-the-art. arXiv.

[46] Zadrozny B., Elkan C. Obtaining calibrated probability estimates from decision trees and naive bayesian classifiers. Proceedings of the ICML.

[47] Zadrozny B., Elkan C. Transforming classifier scores into accurate multiclass probability estimates. Proceedings of the KDD.

[48] Platt J. (1999). Probabilistic outputs for support vector machines and comparisons to regularized likelihood methods. Adv. Large Margin Classif..

[49] Pereyra G., Tucker G., Chorowski J., Kaiser Ł., Hinton G. (2017). Regularizing neural networks by penalizing confident output distributions. arXiv.

[50] Liang G., Zhang Y., Wang X., Jacobs N. (2020). Improved trainable calibration method for neural networks on medical imaging classification. arXiv.

[51] Kumar A., Sarawagi S., Jain U. Trainable calibration measures for neural networks from kernel mean embeddings. Proceedings of the ICML.

[52] Bohdal O., Yang Y., Hospedales T. (2023). Meta-Calibration: Learning of Model Calibration Using Differentiable Expected Calibration Error. Trans. Mach. Learn. Res..

[53] Blundell C., Cornebise J., Kavukcuoglu K., Wierstra D. Weight uncertainty in neural network. Proceedings of the ICML.

[54] Lakshminarayanan B., Pritzel A., Blundell C. Simple and scalable predictive uncertainty estimation using deep ensembles. Proceedings of the NeurIPS.

[55] Gal Y., Ghahramani Z. Dropout as a bayesian approximation: Representing model uncertainty in deep learning. Proceedings of the ICML.

[56] Jang E., Gu S., Poole B. Categorical Reparametrization with Gumble-Softmax. Proceedings of the ICLR.

[57] Zhang J., Kailkhura B., Han T.Y.J. Mix-n-match: Ensemble and compositional methods for uncertainty calibration in deep learning. Proceedings of the ICML.

[58] Laves M.H., Ihler S., Kortmann K.P., Ortmaier T. (2019). Well-calibrated model uncertainty with temperature scaling for dropout variational inference. arXiv.

[59] Naeini M.P., Cooper G., Hauskrecht M. Obtaining well calibrated probabilities using bayesian binning. Proceedings of the AAAI.

[60] Gao C., Li S., Lei W., Chen J., Li B., Jiang P., He X., Mao J., Chua T.S. KuaiRec: A Fully-observed Dataset and Insights for Evaluating Recommender Systems. Proceedings of the CIKM.

[61] Chen J., Dong H., Qiu Y., He X., Xin X., Chen L., Lin G., Yang K. AutoDebias: Learning to Debias for Recommendation. Proceedings of the SIGIR.

[62] Li H., Wu K., Zheng C., Xiao Y., Wang H., Geng Z., Feng F., He X., Wu P. Removing Hidden Confounding in Recommendation: A Unified Multi-Task Learning Approach. Proceedings of the NeurIPS.

[63] Li H., Xiao Y., Zheng C., Wu P. Balancing Unobserved Confounding with a Few Unbiased Ratings in Debiased Recommendations. Proceedings of the WWW.

[64] Koren Y., Bell R., Volinsky C. (2009). Matrix factorization techniques for recommender systems. Computer.

[65] Swaminathan A., Joachims T. The Self-Normalized Estimator for Counterfactual Learning. Proceedings of the NeurIPS.

[66] Guo S., Zou L., Liu Y., Ye W., Cheng S., Wang S., Chen H., Yin D., Chang Y. Enhanced Doubly Robust Learning for Debiasing Post-Click Conversion Rate Estimation. Proceedings of the SIGIR.

[67] Dai Q., Li H., Wu P., Dong Z., Zhou X.H., Zhang R., Zhang R., Sun J. A generalized doubly robust learning framework for debiasing post-click conversion rate prediction. Proceedings of the KDD.

[68] Li H., Xiao Y., Zheng C., Wu P., Cui P. Propensity Matters: Measuring and Enhancing Balancing for Recommendation. Proceedings of the ICML.

[69] Bradley A.P. (1997). The use of the area under the ROC curve in the evaluation of machine learning algorithms. Pattern Recognit..

[70] Järvelin K., Kekäläinen J. (2002). Cumulated gain-based evaluation of IR techniques. ACM Trans. Inf. Syst. (TOIS).

[71] Lipton Z.C., Elkan C., Naryanaswamy B. (2014). Optimal thresholding of classifiers to maximize F1 measure. Proceedings of the Machine Learning and Knowledge Discovery in Databases: European Conference, ECML PKDD 2014.

